# *Rauvolfia vomitoria* phenol extract relieves pentylenetetrazol-induced seizures in Swiss mice and protects some temporal lobe structures

**DOI:** 10.1186/s42494-024-00183-2

**Published:** 2024-11-04

**Authors:** Moses B. Ekong, Okokon O. Bassey, Deborah I. Ebeh, Godslove D. Usukuma, Darlington C. Samuel, Rosemary B. Bassey, Aniekan I. Peter, Christopher C. Mbadugha, Jude E. Okokon, Monday I. Akpanabiatu

**Affiliations:** 1 Department of Anatomy, Faculty of Basic Medical Sciences, University of Uyo, PMB 1017, Uyo, Nigeria; 2 Department of Science Education, Donald and Barbara Zucker School of Medicine at Hofstra/ Northwell, Hempstead, NY 11549 USA; 3 Department of Pharmacology and Toxicology, Faculty of Pharmacy, University of Uyo, PMB 1017, Uyo, Nigeria; 4 Department of Biochemistry, Faculty of Sciences, University of Uyo, PMB 1017, Uyo, Nigeria

**Keywords:** Seizure, Cognitive behaviour, Neurochemicals, Temporal lobe, Sodium valproate, Swiss mice

## Abstract

**Background:**

*Rauvolfia vomitoria* (*R. vomitoria*) is a plant of economic importance due to its diverse ethnomedicinal properties, including the anticonvulsant effect. In this study, we studied the antiseizure and neuroprotective potentials of *R. vomitoria* extracts against pentylenetetrazol (PTZ)-induced kindling.

**Methods:**

Twenty-five adult Swiss mice (25–30 g) were assigned to five groups (*n* = 5): control group, PTZ treatment group, and PTZ treatment after receiving oral *R. vomitoria* crude extract (100 mg/kg), *R. vomitoria* phenol extract (50 mg/kg) or sodium valproate (15 mg/kg) every 48 h for 28 days. Seizure scores, cognitive behavioral tests including novel object test, Y-maze test, and the elevated plus maze test, as well as brain neurochemicals and histomorphology studies, were performed.

**Results:**

Compared with the control group, the PTZ group showed comparable body weight and durations in closed and open arms (*P* > 0.05), but preference for familiar objects, significant (*P* < 0.05) spontaneous alternation, increased monoamine oxidase activity and nitric oxide level, and Nissl chromatolysis in the temporal lobe structures including the cortex, hippocampus, and amygdala. *R. vomitoria* phenol extract pretreatment significantly (*P* < 0.05) reduced seizures, prevented adverse cognitive behaviors, decreased the nitric oxide level, and reduced the temporal lobe Nissl chromatolysis compared with the *R. vomitoria* crude extract pretreatment group and the sodium valproate pretreatment groups.

**Conclusions:**

Thus, *R. vomitoria* phenol extract showed promising results against seizures and potential for general brain protection, suggesting that the anticonvulsant property of *R. vomitoria* may be attributed to its phenol constituent. More studies are needed to delineate the mechanisms of its action.

## Background

Epilepsy is a known neurological condition with seizures being its most acknowledged presentation [1, 2]. Unconsciousness and cognitive deficits are also presentations of epilepsy, either stand-alone or as a manifestation of the seizure [2, 3]. Imbalance of neurochemicals in the brain, especially deficiency in gamma amino butyric acid and increase of glutamate, result in neuronal excitability, which manifests as seizures [4, 5]. Chronic neuronal excitability results in excitotoxicity and associated neuronal damage [5], as reported in temporal lobe epilepsy [6].

The management of epilepsy is increasingly a challenge due to prolonged treatment, resistance to some anti seizure medications, and adverse outcomes in patients [7–9]. To overcome some, if not all, of these issues, alternatives such as herbal extracts [10–12] may be essential since they form the ethnomedicinal practice of society. However, their preclinical evaluations are yet to be fully validated. One such plant is *Rauvolfia vomitoria* (*R. vomitoria*), a plant of economic importance with diverse ethnomedicinal properties including antioxidant, anticonvulsant, and sedative effects due to its abundant phytochemicals [10, 13–15]. One such is polyphenols, which protect neurons against oxidative stress and inflammation [16, 17] and are reported to be protective against neurodegenerative diseases [18].

Oxidative stress is a cause of neuronal toxicity and is also implicated in epilepsy [19, 20]. Prevention or amelioration of oxidative stress can counteract the neurotoxicity [16, 17], leading to neuron protection. This study was set out to study the effects of *R. vomitoria* extracts on epilepsy in pentylenetetrazol (PTZ)-kindled mice.

## Methods

### Preparation of plant material

*R. vomitoria* roots were obtained from a farm in Ekpene Obo in Esit Eket Local Government Area of Akwa Ibom State, Nigeria. The *R. vomitoria* plant was verified at the Department of Botany and Ecological Studies, University of Uyo, with specimen number UUPH 6(c) deposited at the herbarium unit.

The cambium of the roots of *R. vomitoria* was air-dried, broken into small pieces, and ground to a fine powder. The ground *R. vomitoria* cambium was macerated in 80% ethyl alcohol, and the crude extract was concentrated at 45 ℃ in a water bath.

### Phytochemical screening and analyses

The *R. vomitoria* crude extract was qualitatively and quantitatively screened for phytochemicals as described by Harborne [21], Soforowa [22], and Trease and Evans [23]. The protocols for these phytochemical analyses were carried out in a biochemistry laboratory.

### Animals

Adult Swiss mice (25–30 g) were obtained from the Faculty’s Animal House, University of Uyo. A total of 25 mice were acclimatized for 14 days and randomly assigned to five groups (*n* = 5 mice in each group). In group 1 (control group), the mice received normal saline (5 ml/kg body weight, p.o.), while mice in groups 2–5 were administered with PTZ (30 mg/kg, intraperitoneally, Batch number: MKCM4261, Sigma-Aldrich, USA) 30 min after receiving normal saline (5 ml/kg body weight, p.o.), *R. vomitoria* crude extract (100 mg/kg), *R. vomitoria* phenol extract (50 mg/kg), or sodium valproate (15 mg/kg, Batch number: 677289, Sanofi-Aventis, Riells, Spain) for 28 alternate days, respectively. Sodium valproate was used as a standard antiepileptic and a positive control.

The mice were housed in well-ventilated standard animal cages at room temperature of 25–28 ℃ under a 12:12 h light/dark cycle, with *ad libitum* access to standard chow pellet and water. Animal experimental procedures were conducted following the United States National Institute of Health guidelines for laboratory animal use [24], and were approved by the Faculty of Basic Medical Sciences Ethical Committee, University of Uyo (approval number: UU/FBMSREC/2022/001).

### Animal kindling

PTZ was used to develop the mouse kindling model. The chemical was dissolved in normal saline, and a single dose of 30 mg/kg was administered to each mouse every 48 h. A total of 14 doses were administered. Seizures in each mouse were scored 0–5 following the Racine scale. A mouse with Racine scores of 4–5 (tonic-clonic seizure) on three consecutive administrations was regarded as kindled [25].

### Cognitive behavioral tests

The cognitive-behavioral tests were carried out on days 20–22. Mice were acclimatized in the behavioral test room for an hour before the test. Each behavioral test protocol was video-recorded and the parameters were manually scored.

### Novel object recognition test

The novel object recognition test was performed in an open field maze on days 20–22 to assess learning and memory. The maze was an open field of 100 × 100 × 50 cm^3^. The test involved three objects of equal size but of different shapes (two similar and one novel). The mice were habituated to explore the empty maze for five minutes on day 20. One day 21, the mice explored two identical objects in the same maze for 10 min. On day 22, a novel object was put in the maze and the mice were allowed to explore the three objects for 10 min. The exploration times for the familiar and novel objects were recorded. The discrimination index was calculated as follows:

$$\:DI=\frac{T\left(new\right)-T\left(old\right)}{T\left(total\right)}$$

Where *DI* represents discrimination index; *T*(new) represents the time spent exploring the novel object; *T*(old) represents the time spent exploring the familiar objects; and *T*(total) equals the total time spent on all objects. The maze was cleaned with ethyl alcohol between trials [26, 27].

### Y-maze test

The Y-maze test was performed on day 21. The spontaneous alternation behaviour was assessed to reflect short-term working memory [28]. The Y-maze consisted of three identical arms (40 × 30 × 10 cm, width × length × height), mounted in the shape of a “Y”. A mouse was introduced into the start arm to explore the maze for 8 min, and the spontaneous alternations and total arm entries were recorded. An arm entry was accepted when the four limbs of the mouse were completely within it, and a spontaneous alternation was considered when the mouse entered three different arms. The alternation percentage was calculated using the formula: successive alternation sets/(total number of arm entries – 2) × 100%. After each trial, the maze was wiped with 70% ethyl alcohol [29].

### Elevated plus maze test

Elevated plus maze test was performed on day 21 to assess anxiety-related behavior. The maze was cross-shaped consisting of two open arms (100 × 10 cm), two closed arms (100 × 10 × 40 cm) and a central area (10 × 10 cm) [30]. Each mouse was placed in the central area, and explored there for 5 min. Durations in open and closed arms, as well as grooming and rearing behaviors were scored. The maze was wiped with 70% ethyl alcohol between trials.

### Biochemical and histological assays

After completion of the behavioral tests, the mice were anesthetized by intraperitoneal injection of ketamine hydrochloride (50 mg/kg body weight, Rotex Medica, Germany, Batch number: 20KA03) and sacrificed by transcardial perfusion with cold phosphate-buffered saline. The brain was removed. One hemisphere was fixed in 10% buffered formalin for histology and the other hemisphere was homogenized for biochemical assay.

For biochemical assay, the brain tissue was homogenized in 0.1 M phosphate buffer (pH 7.2), and the homogenates were centrifuged at 3000 rpm for 15 min. Aliquots of each supernatant were used to determine the activity of monoamine oxidase (MAO) and glutamate dehydrogenase (GDH) activities as well as the level of nitric oxide [31, 32].

For histological assay, six serial Sect. (10-µm thick) of the forebrain region containing temporal cortex, dorsal hippocampus, and amygdala from each animal (six per slide) were processed for Nissl staining using Cresyl fast violet stain.

### Statistical analysis

GraphPad Prism (version 10.2.3) was used for all analyses. As the sample size was small, the data were tested for normality and variance homogeneity. To test for normality, the Kolmogorov-Smirnov test was applied, to determine if the data followed a Gaussian distribution. The Brown-Forsythe test was applied to test for variance homogeneity, with no significant probability level. Comparisons between groups were performed with one-way analysis of variance followed by Tukey’s post-hoc test. *P* < 0.05 was considered as statistically significant. Data are presented as the mean ± standard error of the mean.

## Results

### Phytochemicals of *R. vomitoria* root bark

Phytochemical screening of *R. vomitoria* root bark identified the most abundant and active constituents. Qualitative analyses showed that alkaloids, flavonoids, phenols, saponins, terpenoids, and tannins were constituents of *R. vomitoria* root bark.

**Fig. 1:**
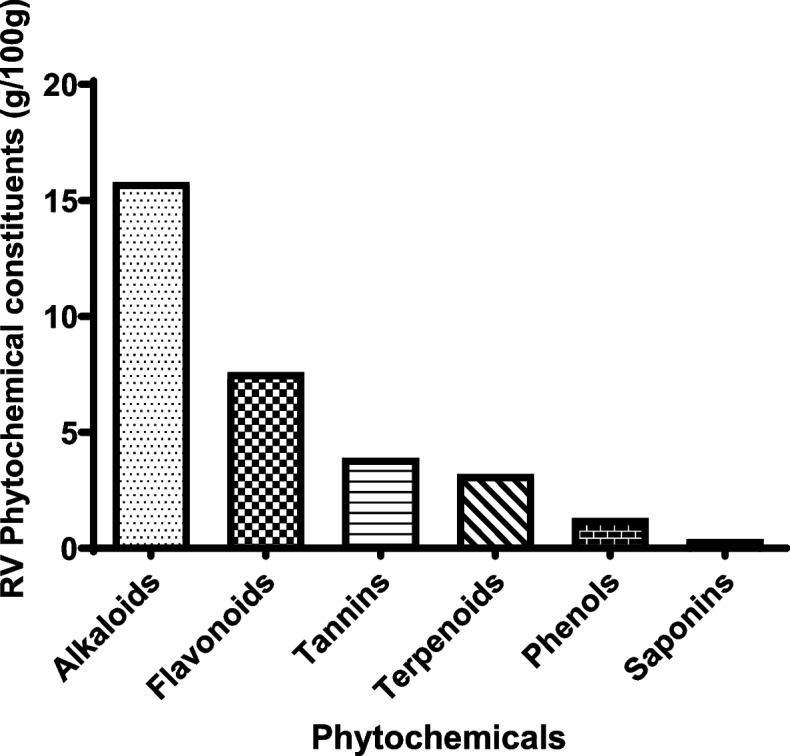
Quantitative analysis of phytochemical compositions of *R. vomitoria* root bark

Alkaloids (15.80 g per 100 g) were the most abundant constituents of *R. vomitoria* root bark. Phytochemicals with moderate concentrations included flavonoids (7.60 g per 100 g), tannins (3.90 g per 100 g), and terpenoids (3.90 g per 100 g). However, phenols (1.30 g per 100 g) and saponins (0.40 g per 100 g) were obtained in small quantities (Fig. 1).

### Body weight assessments

**Fig. 2:**
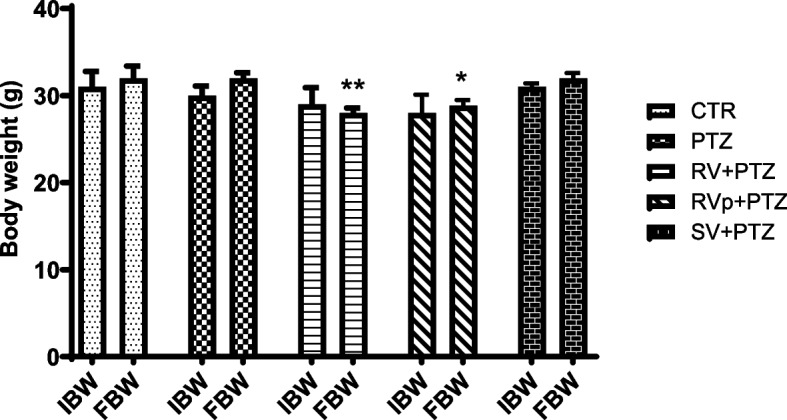
Mouse body weights at the start of study and at sacrifice. One-way analysis of variance followed by Tukey's multiple comparison test; *n* = 5 mice in each group. IBW, initial body weight; FBW, final body weight; PTZ, pentylenetetrazol; RV, *R. vomitoria* crude extract; RVp, *R. vomitoria* phenol extract; SV, sodium valproate. *P < 0.05 vs SV+PTZ group; **P < 0.01 vs CTR, PTZ, SV+PTZ groups

The initial body weight did not significantly differ among groups (*F* [4, 20] = 0.72, *P* = 0.5911). At sacrifice, the body weight significantly differed (*F* [4, 20] = 6.9, *P* = 0.0007). No difference was observed between the PTZ group and the control group, but there was a significant body weight loss in the *R. vomitoria* crude extract pretreatment group compared with the control and PTZ groups, as well as sodium valproate pretreatment group. The *R. vomitoria* phenol extract pretreatment and sodium valproate pretreatment groups showed no significant difference from the control and PTZ groups, but the body weight of the *R. vomitoria* phenol extract pretreatment group was significantly less than the sodium valproate pretreatment group (Fig. 2).

### Seizure scores

**Fig. 3:**
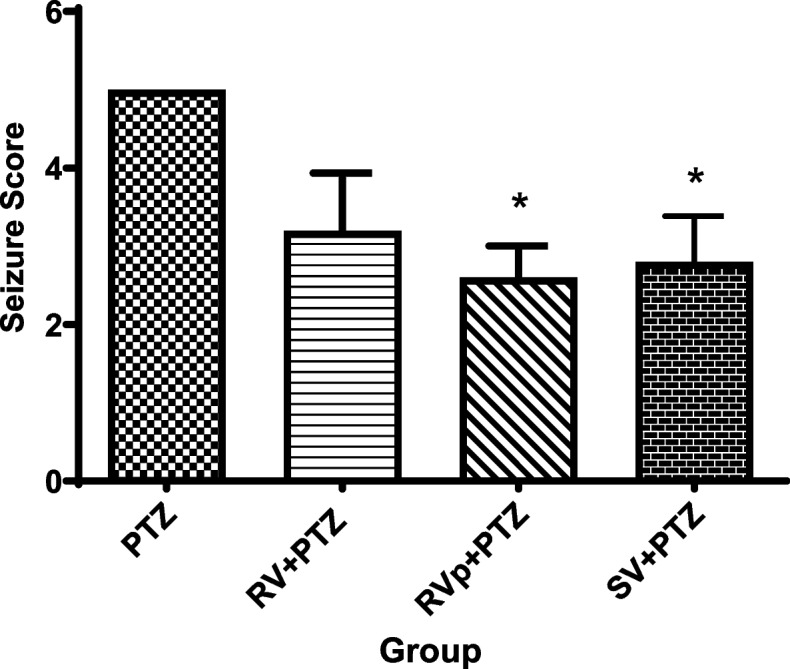
Racine seizure scores in the groups. One-way analysis of variance followed by Tukey's Multiple Comparison Test; *n* = 5; PTZ, pentylenetetrazol; RV, *R. vomitoria* crude extract; RVp, *R. vomitoria* phenol extract; SV, sodium valproate. *P < 0.05 vs the PTZ group

The final seizure score of the PTZ group was significantly higher (*F* [3, 16] = 4.6, *P* = 0.017) compared with the *R. vomitoria* phenol extract pretreatment or sodium valproate pretreatment group. The *R. vomitoria* crude extract pretreatment group showed no significant difference (*P* < 0.05) in the final seizure scores compared with the PTZ group (Fig. 3).

### Novel object recognition and discriminative indices

**Fig. 4:**
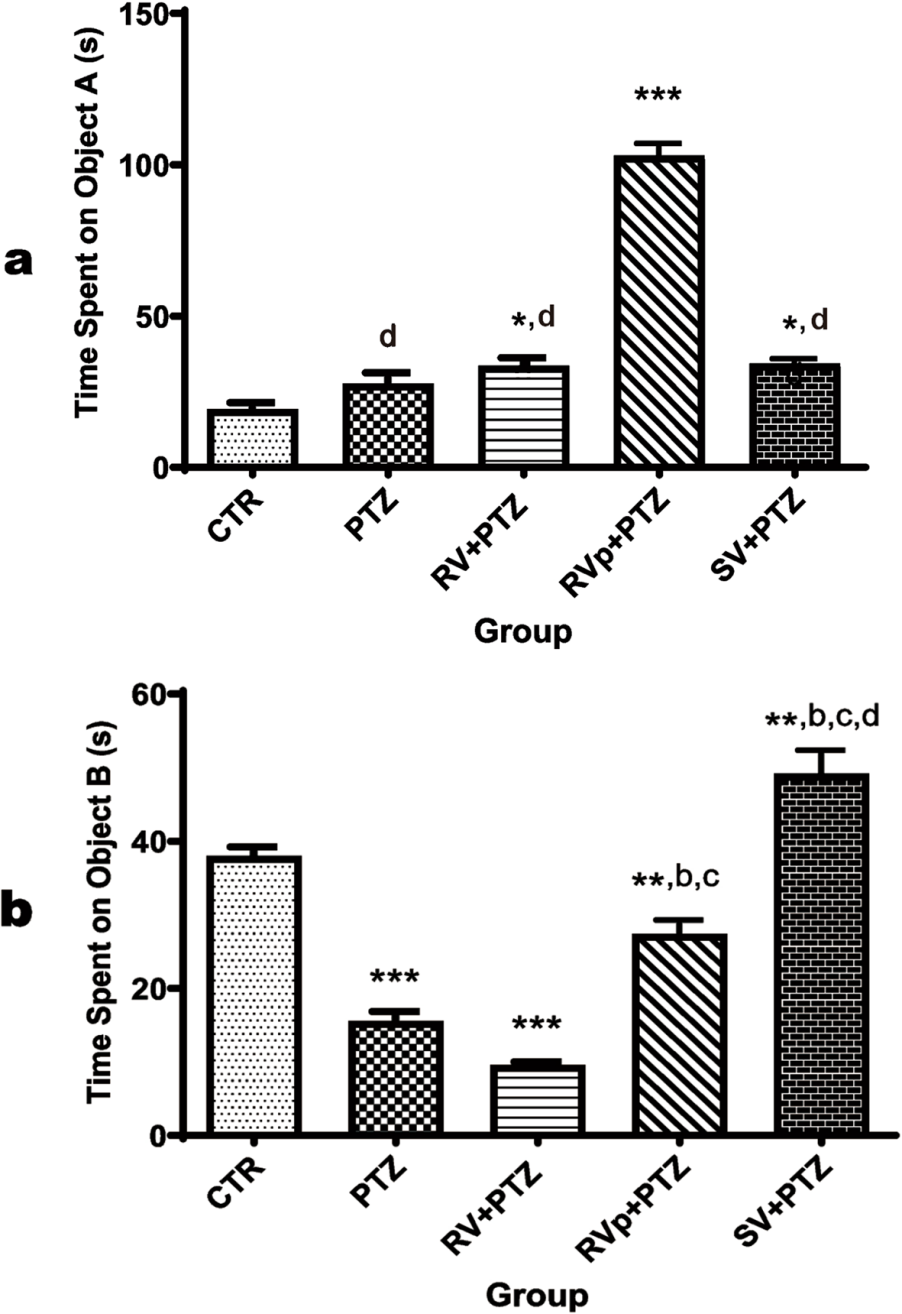
Novel object recognition of mice in the experimental groups. One-way analysis of variance followed by Tukey's Multiple Comparison Test; *n* = 5; PTZ, pentylenetetrazol; RV, *R. vomitoria* crude extract; RVp, *R. vomitoria* phenol extract; SV, sodium valproate. *P< 0.05 vs the CTR group; **P< 0.01 vs the CTR group;***P< 0.001 vs the CTR group; ^b^*P*< 0.05 vs the PTZ group; ^c^*P *< 0.05 vs the RV+PTZ group; ^d^*P *< 0.05 vs the RVp+PTZ group

No difference (*F* [4, 20] = 134.4, *P* < 0.0001) was found for familiar object preference between the PTZ group and the control group. However, mice in the *R. vomitoria* crude extract, phenol extract, or sodium valproate pretreatment group spent significantly more time with the familiar object compared to the control. The *R. vomitoria* phenol extract group spent significantly more time with the familiar object compared to other test groups (Fig. 4a).

Compared to the control group, mice in the PTZ-only group, the *R. vomitoria* crude extract pre-treatment group, and the phenol extract pretreatment group had significantly lower preference for the novel object (*F* [4, 20] = 76.99, *P* < 0.0001). However, mice in the sodium valproate pretreatment group spent significantly more time with the novel object compared to the controls. Additionally, mice in the *R. vomitoria* phenol extract pretreatment group or sodium valproate pretreatment group also spent significantly more time with the novel object compared to the PTZ and *R. vomitoria* crude extract groups (Fig. 4b).

The discrimination index was positive in the control (0.3) and sodium valproate (0.2) groups, but negative in the PTZ (-0.3), *R. vomitoria* crude extract pretreatment (-0.5), and *R. vomitoria* phenol extract pretreatment (-0.6) groups.

### Spontaneous alternation

**Fig. 5:**
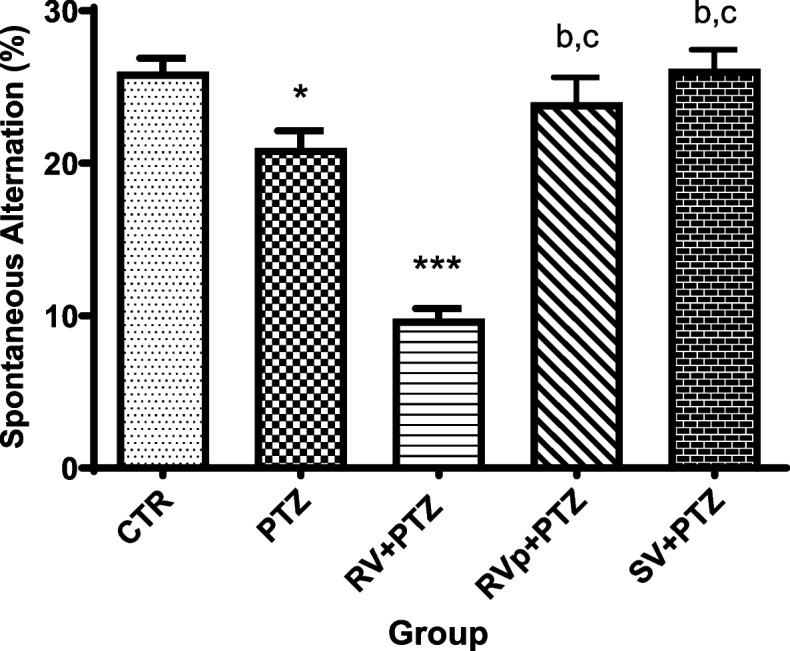
Spontaneous alternations of mice. One-way analysis of variance followed by Tukey's Multiple Comparison Test;* n* = 5; PTZ, pentylenetetrazol; RV, *R. vomitoria* crude extract; RVp, *R. vomitoria* phenol extract; SV, sodium valproate. *P< 0.05 vs the CTR group; ***P < 0.001 vs the CTR group; ^b^*P *< 0.05 vs the PTZ group; ^c^*P *< 0.05 vs the RV+PTZ group

Mice in the PTZ group and the *R. vomitoria* crude extract pretreatment group showed significantly decreased spontaneous alternation percentages compared with the control group. The spontaneous alternation percentages in the *R. vomitoria* phenol extract pretreatment and the sodium valproate pre-treatment groups did not significantly differ from that in the control group, but were significantly higher than those in the PTZ group and the *R. vomitoria* crude extract pretreatment group (Fig. 5).

### Elevated plus maze

**Fig. 6:**
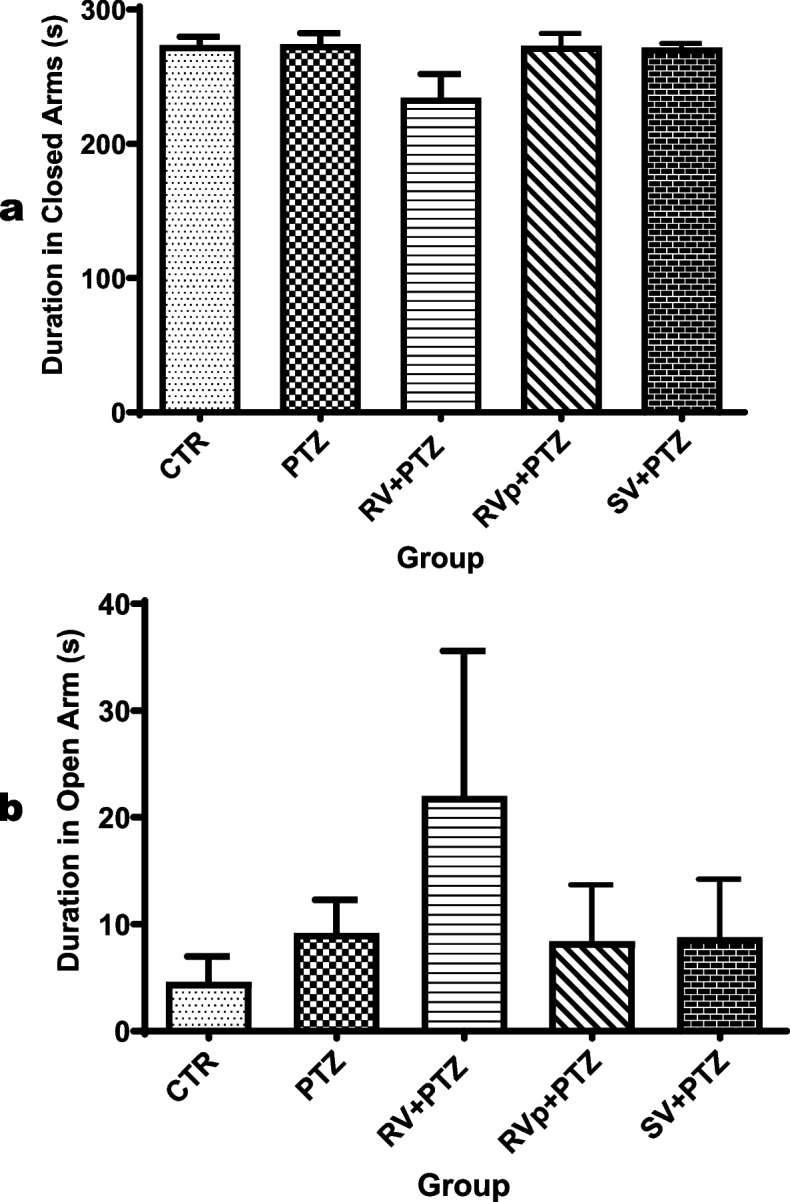
Anxiety-like behavior in the elevated plus maze. One-way analysis of variance followed by Tukey's Multiple Comparison Test; *n* = 5; PTZ, pentylenetetrazol; RV, *R. vomitoria* crude extract; RVp, *R. vomitoria* phenol extract; SV, sodium valproate. There was no significant difference among the groups

The durations in the open arms (*F* [4, 20] = 2.9, *P* = 0.0461) and closed arms (*F* [4, 20] = 0.86, *P* = 0.5063) in the PTZ group, *R. vomitoria* crude extract pretreatment group, phenol extract pretreatment group, or sodium valproate pretreatment group did not significantly differ from the control group (Fig. 6a, b).

### Brain biomolecules

**Fig. 7:**
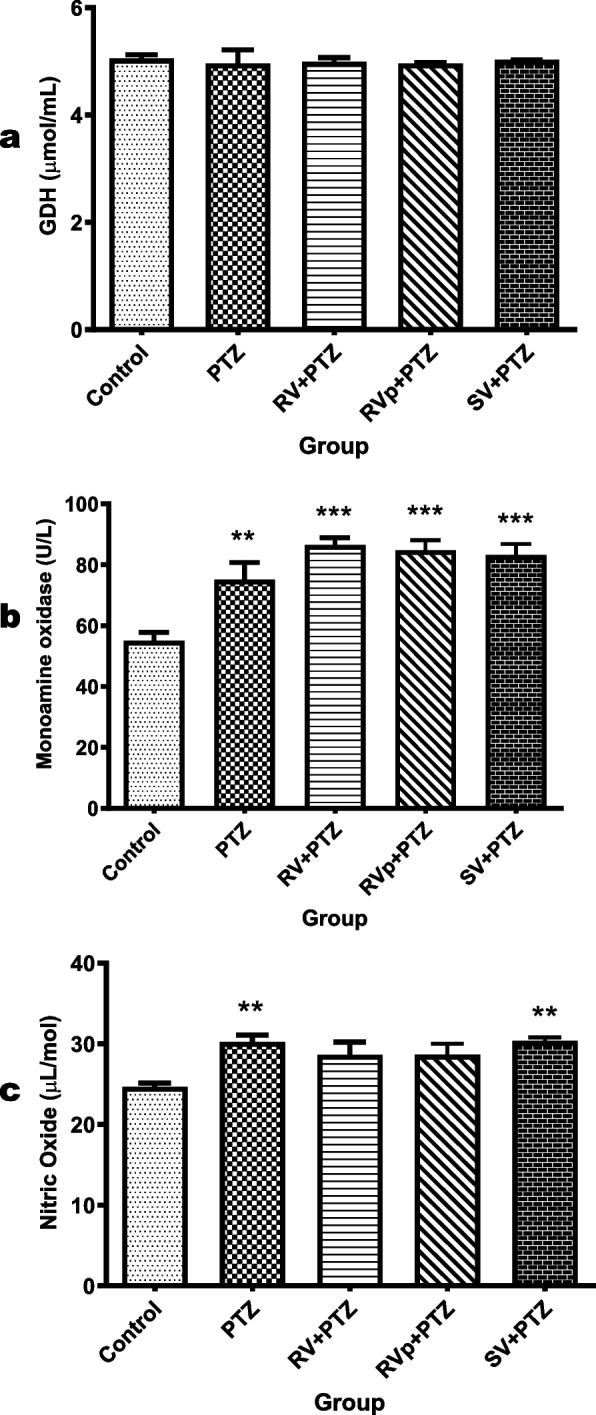
Biomoelcules in the mouse brain in the experimental groups. **a** GDH (glutamate dehydrogenase) activities in the brain.** b** MAO activities in the brain. **c** NO levels in the brain. One-way analysis of variance followed by Tukey's Multiple Comparison Test; *n* = 5; PTZ, pentylenetetrazol; RV, *R. vomitoria* crude extract; RVp, *R. vomitoria* phenol extract; SV, sodium valproate. **P < 0.01 vs the CTR group; ***P < 0.001 vs the CTR group

There was no significant difference (*F* [4, 20] = 0.11, *P* = 0.9785) in the brain GDH activity among the groups (Fig. 7a).

The brain MAO activity in the PTZ group, *R. vomitoria* crude extract pretreatment group, phenol extract pretreatment group, and sodium valproate pretreatment group was significantly higher (*F* [4, 20] = 4.9, *P* = 0.0064) compared with that in the control group (Fig. 7b).

The brain nitric oxide levels in the PTZ group and the sodium valproate pretreatment group were significantly higher (*F* [4, 20] = 12, *P* < 0.0001) compared to that in the control group. However, the brain nitric oxide levels in the *R. vomitoria* crude extract pretreatment group and phenol extract pretreatment group were not significantly different from those in the control group and the other test groups (Fig. 7c).

### Brain histology

#### Hippocampus

**Fig. 8:**
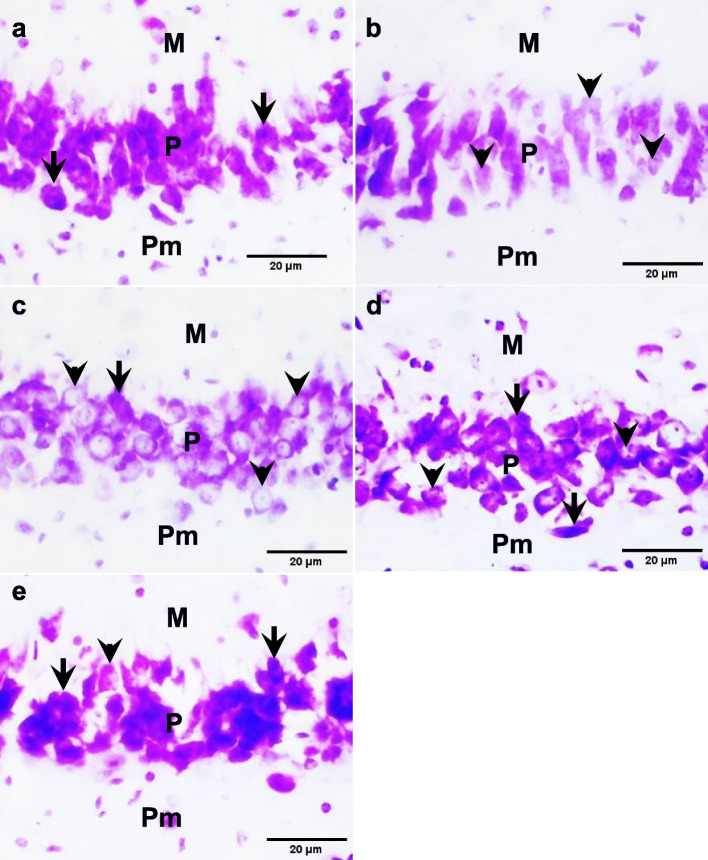
Nissl staining in the hippocampal CA3 region. **a** Well-expressed Nissl in the control group. **b **Some less-expressed Nissl in the PTZ group. **c** Moderately-expressed Nissl in the* R. vomitoria* crude extract pretreatment group. **d **Well-expressed Nissl in the *R. vomitoria* phenol extract pretreatment group. **e** Well-expressed Nissl in the sodium valproate pretreatment group. M, molecular layer; P, pyramidal layer; Pm, polymorphic layer. Black arrows indicate dense staining, and black arrowheads indicate weak staining. Cresyl violet, ×400

Histological sections of the hippocampal CA3 region showed Nissl staining in the molecular, pyramidal, and polymorphic layers in the control group. The PTZ group and the *R. vomitoria* crude extract pretreatment group showed less Nissl staining in the cortical layers compared to the control group. The *R. vomitoria* phenol extract pretreatment group showed moderate Nissl staining in the cortical layers compared to the control. The sodium valproate pretreatment group showed dense Nissl staining in the cortical layers compared to the control group (Fig. 8).

#### Amygdala

**Fig. 9:**
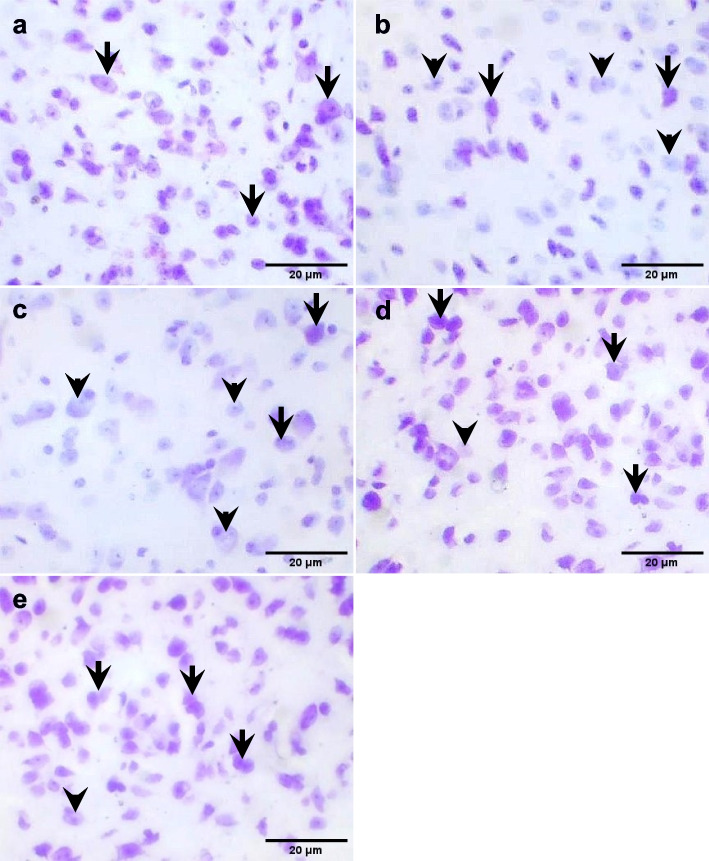
Nissl staining in the amygdala. **a** Well-expressed Nissl staining in the control group. **b** Moderate-expressed Nissl staining in the *R. vomitoria* crude extract pretreatment group. **c** Less-expressed Nissl staining in the PTZ group. **d** Well-expressed Nissl staining in the *R. vomitoria* phenol extract pretreatment group. **e **Well-expressed Nissl in the sodium valproate pretreatment group. Black arrows indicate dense staining, and black arrowheads indicate weak staining. Cresyl violet, ×400

Histological sections of the centromedial area of the amygdala showed numerous cells of varying sizes with Nissl staining signals in the control group. the PTZ group and the *R. vomitoria* crude extract pretreatment group showed less Nissl staining. The *R. vomitoria* phenol pretreatment group and the sodium valproate pretreatment group showed comparable Nissl staining signals to the control group (Fig. 9).

#### Temporal cortex

**Fig. 10:**
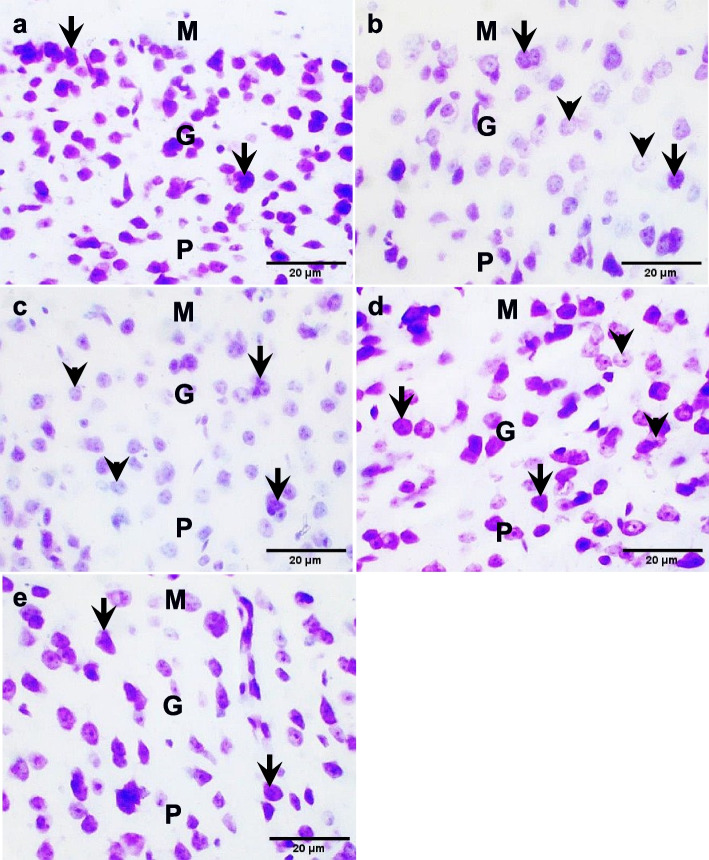
Nissl staining in the temporal cortical layers.** a** Nissl staining in the control group. **b** Weak Nissl staining in the PTZ group. **C **Weak Nissl staining in the *R. vomitoria* crude extract pretreatment group. **d **Well-expressed Nissl in the *R. vomitoria* phenol extract pretreatment group. **e** Well-expressed Nissl in the sodium valproate pretreatment group. M, molecular layer; P, pyramidal layer; Pm, polymorphic layer. Black arrows indicate dense staining, and black arrowheads indicate weak staining. Cresyl violet, ×400

Histological sections of the temporal cortex showed less Nissl staining in numerous temporal cortical cells of the PTZ group and the *R. vomitoria* crude extract pretreatment group compared to the control group. Nissl was well-expressed in numerous temporal cortical cells of The *R. vomitoria* phenol extract pretreatment group and the sodium valproate pretreatment group showed comparable Nissl staining signals compared to the control group (Fig. 10).

## Discussion

This present study investigated the effects of root bark extracts of *R. vomitoria* on PTZ-induced epilepsy and compared their effects to sodium valproate, a standard antiseizure medication. The results showed that the *R. vomitoria* phenol extract improved spontaneous alternation, preference for the novel object and MAO activity, decreased the nitric oxide level, and improved Nissl staining signals in the hippocampus, amygdala, and temporal cortex.

Phytochemicals are the basis of various pharmacological effects of plants [33, 34], whose specific action is determined by their compositions. In this study, we identified the following phytochemicals of *R. vomitoria* root bark: alkaloids, flavonoids, phenols, saponins, terpenoids, and tannins. Consistent with previous studies [14, 35], alkaloids were the most concentrated constituents of *R. vomitoria* root bark. Flavonoids and phenols had comparable amounts. Phenols are antioxidants [15, 18]. Anticonvulsant activity of *R. vomitoria* has been reported [10], but the contributing compound is yet to be identified.

Body weight is usually used for the assessment of the overall well-being of individuals. In the present study, the mice in various groups had the same baseline body weights. At the end of the study, the final body weight did not differ significantly between the PTZ group and the control group, indicating that body weight was not affected by PTZ kindling. However, mice in the *R. vomitoria* crude extract pre-treatment group showed weight loss, which is consistent with reported implication of *R. vomitoria* in body weight loss [36, 37] that is attributed to the side effects such as sedation and loss of appetite [13, 36].

The *R. vomitoria* phenol extract pretreatment group and the sodium valproate pretreatment group showed no significant difference in the final body weight compared with the control group, which indicated that *R. vomitoria* phenol extract did not cause weight loss. Sodium valproate has been reported to enhance body weight [38–40]; the lack of significance in the body weight difference may be due to the administration schedule.

One method of inducing experimental epilepsy in animal models is through the administration of PTZ at sub-convulsive doses every 48 h [25]. Epilepsy is usually measured as seizure scores following the Racine scale [41, 42]. The PTZ group showed a seizure score of 5, indicating kindling in animals, since scores of 4 and 5 indicate tonic-clonic seizures [25]. PTZ blocks the gamma-aminobutyric acid type-A receptor [43] and may up-regulate *N*-methyl-*D*-aspartate receptors [44]. These actions usually lead to seizures.

The final seizure score of the *R. vomitoria* crude extract pretreatment group was not different from that of the PTZ group, indicating that crude extract may limit the antiseizure activities of the plant. *R. vomitoria* crude extract is reported to have antisiezure action [10], but the extent may be due to the general constituents.

The final seizure scores of the *R. vomitoria* phenol extract pretreatment group and the sodium valproate pretreatment group were significantly less than the PTZ group, indicating protection against seizures. Most antiseizure medications, including sodium valproate, act at the gamma-aminobutyric acid type-A receptor, leading to enhancement of brain gamma-aminobutyric acid and the attenuation of convulsions [8, 45, 46]. The *R. vomitoria* phenol extract may act through the GABAergic pathway, suppressing the action of PTZ on the gamma aminobutyric acid receptor. Ebuehi and Aleshinloye [47] reported enhanced GABA activity of *R. vomitoria* extract. The present result also supports the finding by Singh et al. [48], who reported that flavonoids, which are major phenols and present in *R. vomitoria*, enhance gamma-aminobutyric acid transmission by stimulating the gamma-aminobutyric acid receptors and voltage-gated ion channels [49].

Cognitive and emotional functions are fundamental and complicated processes [50] that can be impaired in epilepsy [51, 52]. These functions can be tested in animal models using novel object recognition test, spontaneous alternation test, and elevated plus maze test, among others. The novel-object recognition evaluates learning and memory by assessing the preference for a novel, rather than a familiar object [26, 27]. The cerebral cortex and hippocampus play a role in recognition memory [53–55]. The discrimination index, analogous to recognition memory in humans [27], was assessed in the present study. The PTZ group spent more time on the familiar objects than the novel object, resulting in a negative discrimination index reflecting a preference for familiar objects.

Mice in the *R. vomitoria* crude extract pretreatment group or the phenol extract pretreatment group spent significantly more time with the familiar objects than the novel object, suggesting a preference for the familiar objects and an inability to protect the altered recognition memory [53, 54]. However, the sodium valproate pretreatment group showed preference for the novel object and a positive discrimination index, reflecting protection of this brain function.

Spontaneous alteration test evaluates the spatial working memory function of the hippocampus [30]. In this test, animals exposed to the Y-maze remember a previously visited arm and tend to enter less frequently explored ones [30, 56]. In the present study, there was a significant decrease in spontaneous alternation in the PTZ group, indicating poor spatial working memory. PTZ is reported to cause memory impairment [57, 58]. This result is inconsistent with the study of Lamberty and Klitgaard [59], who reported no difference.

The spontaneous alternation significantly decreased in the *R. vomitoria* crude extract pretreatment group, which was not different from the control and the PTZ groups, suggesting its sedative action on the brain area involved in learning and memory. It is reported that the *R. vomitoria* extract induces sedation [13, 36], with involvement of the brain area for memory consolidation.

There was no significant difference in the spontaneous alternation of the *R. vomitoria* phenol extract pretreatment group and the sodium valproate pretreatment group compared with the control group, but they were significantly higher than those of the PTZ group and the *R. vomitoria* crude extract pretreatment group. These results suggest their enhanced effect on spatial working memory [30].

The elevated plus maze test predicts anxiety-like behaviour in animal models [60]. Aversion to open arms indicates anxiogenicity of the administered substance, while non-aversion suggests anxiolytic effect [60, 61]. In the present study, there was no significant difference in the duration of mice in the open and closed arms between the PTZ group and the control group. This result is inconsistent with Jung et al. [62], who reported that PTZ elicits anxiety.

There was no significant difference in the duration in the open and closed arms of the elevated plus maze in the *R. vomitoria* crude extract pretreatment group or the phenol extract pretreatment group compared to the control and the PTZ groups, suggestive of an anxiogenic effect of the extracts. The elevated plus-maze test measures anxiety-like activities in animal models [63].

Epilepsy disrupts the electrical neuronal signal due to diminished GABA or elevated glutamate neurotransmitter [64]. Glutamate is the main excitatory neurotransmitter, and its excessive level enhances excitoxicity and epilepsy [5]. GDH regulates the glutamate level by catalyzing glutamate to alpha-ketoglutarate [65]. In the present study, no significant difference in brain GDH activity was observed between the PTZ group and the control group, suggesting that PTZ did not increase glutamate in this study.

There was no significant difference in the brain GDH activity between the *R. vomitoria* crude extract pretreatment group, *R. vomitoria* phenol extract pretreatment group or sodium valproate pretreatment group and the control or PTZ groups, excluding the involvement of GDH pathway. *R. vomitoria* extract boost glutamate levels [47] but this enzyme may not be involved.

MAO regulates the actions of dopamine, serotonin, epinephrine, and norepinephrine, among others [66], thereby modulating the behavior [66, 67]. In the present study, there was significantly higher brain MAO activity in the PTZ group compared with the control, suggesting reduced brain monoamine neurotransmitter levels. The reduced monoamine levels result in behavioral impairment [66, 67], which may explain the altered cognitive ability in seizures.

There was significantly higher MAO activity in the *R. vomitoria* crude extract pretreatment group, the phenol extract pretreatment group, and the sodium valproate pretreatment group, compared with the controls. Oyeniran et al. [18] reported that *R. vomitoria* phenol extracts can reduce MAO activity, but this was not the case in the present study.

Nitric oxide is an endothelium-derived relaxing factor that is endogenously synthesized and mediates important physiological processes, especially the actions of neurotransmitters [68]. A high nitric oxide level is associated with pathology [69]. In the present study, a significantly higher level of brain nitric oxide was observed in the PTZ group compared with the controls, indicating that PTZ induces adverse brain cell activity. PTZ is reported to increase serum, kidney, and brain levels of nitric oxide [70, 71] and associated oxidative stress, as well as epileptic seizures [71]. However, the *R. vomitoria* crude extract pretreatment group or the phenol extract pretreatment group did not show significantly different nitric oxide level from the control group, suggesting a protective action of these extracts.

A significantly higher brain level of nitric oxide was also observed in the sodium valproate pretreatment group compared with the control group. Valproic acid is known to increase nitric oxide in patients with epilepsy under treatment [72], which also indicates oxidative stress and aligns with the present study.

The temporal lobe is involved in cognitive and emotional functions and is implicated in temporal lobe epilepsy [73]. The temporal lobe comprises the temporal cortex, hippocampus, and amygdala, among others [74]. Here, the hippocampal CA3 region, the centromedial area of the amygdala, and the outer temporal cortical layers showed less Nissl staining in the PTZ group, indicative of its adverse effects on these brain regions. The Nissl bodies represent the granular endoplasmic reticulum in neurons [75] and are also seen in glia [76–78], which are responsible for protein synthesis. These Nissl exhibit chromatolysis as a result of different traumatic exposures, [79, 80] which can be observed in our study.

The Nissl staining in the hippocampal CA3 region, the centromedial area of the amygdala, and the outer temporal cortical layers showed less signals in the *R. vomitoria* crude extract pretreatment group. *R. vomitoria* crude extract has adverse histological effects on different brain regions [11, 12, 36, 37], and as such, it may have disrupted the Nissl substance.

The hippocampal CA3 region, the centromedial area of the amygdala, and the outer temporal cortical layers showed comparable Nissl staining in the *R. vomitoria* phenol extract pretreatment group or the sodium valproate pretreatment group compared to the control group, indicating potential protective effects of the phenol extract and sodium valproate.

The temporal lobe is involved in audition, speech and language recognition, and memory [74]. The decreased seizures upon *R. vomitoria* phenol extract pretreatment may indicate protection of these functions and the associated structures.

### Limitations of the study

This study has limitations. First, this is a preliminary study, as the phenol dosage requires optimization. Second, the effective phenol compound(s) were not identified. Further profiling using gas chromatography/mass spectrometry and high-performance liquid chromatography is needed. Third, although in this study the duration and long-term use of phenols generally did not pose a health challenge, it is possible that the long-term use of *R. vomitoria* phenol extract may show a positive effect. Last, this study did not monitor brain seizure activities in real-time due to the inavailability of appropriate electrophysiology, immunohistochemical and western blot techniques. These analyses could have provided additional data to help us understand the mechanism of the antiseizure activity of *R. vomitoria* phenol extract.

## Conclusions

This study showed that the PTZ-induced seizures and associated cognitive behavioral deficits enhance MAO and nitric oxide activities while altering Nissl staining signals in the hippocampus, amygdala, and temporal cortex. *R. vomitoria* phenol extract significantly decreased seizures and protected against adverse cognitive behaviors and nitric oxide, as well as Nissl staining in the hippocampus, amygdala, and temporal cortex. *R. vomitoria* phenol extract showed potentially promising effects against seizures and general brain protection. However, further investigations are needed to delineate its mechanism of action.

## Data Availability

Not applicable.

## Abbreviations

CA3: Cornu ammonis
CTR: Control
GABA: Gamma aminobutyric acid
GDH: Glutamate dehydrogenase
MAO: Monoamine oxidase
PTZ: Pentylenetetrazol
*R. vomitoria*: *Rauvolfia vomitoria*
RV: *Rauvolfia* *vomitoria* crude extract
RVp: *Rauvolfia vomitoria* phenol extract
